# Dataset on industrial waste compositions in West Kazakhstan and conditions for processing them into construction materials

**DOI:** 10.1016/j.dib.2024.110265

**Published:** 2024-03-10

**Authors:** Saule M. Bazarbayeva

**Affiliations:** Faculty of Architecture and Civil Engineering, L.N. Gumilyov Eurasian National University, 13 Kazhymukan Street, 010005 Astana, Kazakhstan

**Keywords:** Monochromate sludge, Chemical production, Oil industry, Recycling, Disposal technology, Sulphur concrete

## Abstract

The paper presents data on the complex processing of large-tonnage waste from oil and gas industry and chemical production, as well the methods of their utilization and processing.

The data was collected at the industrial sites of the plants in Western Kazakhstan.

During the work, there were studied data on the chemical composition of the waste, properties of the experimental compositions, material structure, and peculiarities of the technological processes of recycling. When processing the data, there were used such physical-chemical and mechanical methods as X-ray structure, mass and IR spectroscopy, radiometric analysis of the experimental samples. The paper provides data on thermodynamic calculations for chemical reactions of interaction between components of the compositions.

It is established that the reaction of interaction of petroleum sulphur with sludge monochromate in the production of polymer concrete significantly reduces their toxicity. On the basis of researching data on the composition and properties of wastes and semi-finished products evidence about the possibility to recycle them into goods with positive ecological and economic effect has been obtained.

The given data on practical methods of complex processing and waste recycling (on the example of Western Kazakhstan), allow systematically solving the problem of solid waste management, realizing the basic ecological principle “waste to income” and may be suitable for similar enterprises in other developing countries.

## Graphical abstract

**Figure:**
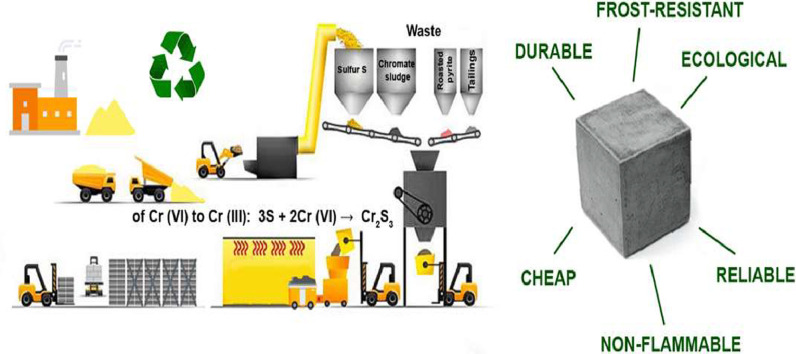

Specifications Table

**Table:** 

Subject	Environmental Science
Specific subject area	Waste Management and Disposal
Data format	Raw, Analyzed
Type of data	Table, Image, Figure
Data collection	Sulphur samples in the form of waste obtained from two deposits were processed by the quartering method. Mass moisture was determined by a method based on the weight determination of weight loss during drying at 70±20°C. Sulphur radioactivity was determined using a dosimeter and a radioactivity counter with an STS-100 sensor. The nature of the interaction of sulphur waste with the components of other dry production waste, as well as the structure of the feedstock and mastics based on them, was studied using IR spectroscopic analysis. The elemental composition was recorded by X-ray spectral analysis by recording topographic maps of the distribution of a chemical element on the surface of a thin section.
Data source location	Zhanazhol and Tengiz oil and gas fields in Western Kazakhstan, Ferroalloy Plant and Chemical Plant industrial sites, YANKA construction firm Western Kazakhstan
Data accessibility	Repository name: Mendeley Data Data identification number: DOI:10.17632/twgctdsk23.3 Direct URL to data: https://data.mendeley.com/datasets/twgctdsk23/3

## 1 Value of the Data

• In West Kazakhstan industrial production are carried out mainly by traditional methods, without the use of waste-free technologies. Sludge reservoirs and tailings ponds of the enterprises of Western Kazakhstan contain millions of tonnes of toxic waste in the form of monochromate sludge of the chemical plant (Aktobe, Western Kazakhstan), pyrite slag - waste from the chemical plant, dry boron-containing waste (Alga, Western Kazakhstan), “tailings” of the ferroalloy plant (Aktobe, Western Kazakhstan). They are replenished at each production cycle.
• Sulphur is produced in Western Kazakhstan, where the oil industry is concentrated, is formed during the oxidative processing of sour gases, is a large-tonnage waste of the oil and gas industry and has a technogenic impact on environmental objects; it accumulates annually in significant quantities and has limited use so far.
• Maintaining economic [1], environmental [2], [3], [4], [5], [6] strategies as well as other [7] aspects concerning the theory and methods of “zero waste”, this work provides presents data on the comprehensive way of disposal of chromium-, boron- and sulphur-containing waste, “tails” of the chemical production, oil and ferroalloy industries in the West of Kazakhstan, which are situated close to each other.
• The proposed data on waste compositions and methods of their processing allow their comprehensive use as raw materials in the production of construction materials and products such as mastic and sulphur concrete, which will solve important economic problems associated with reducing the shortage of natural materials, as well as reducing and disposing of harmful emissions and minimising the impact of hazardous waste on the environment and humans.
• A set of experimental data on the composition of various industrial wastes located in close proximity to each other allows us to identify ways of neutralising their toxicity through fusion and interaction to produce new materials with consumer value. The data presented may be useful to state and local authorities, large industrial enterprises, construction companies, recycling facilities, environmental agencies and researchers in this field.
• The data obtained in this work can be compared with similar data conducted on industrial waste in developing countries. They can be replicated in those industrial regions with relatively similar industries using the proposed methodologies.

## 2 Data Description

The presented dataset shows the chemical composition of wastes from industrial enterprises located in Western Kazakhstan, composition and properties of experimental compositions, structure of materials, peculiarities of technological processes, the data of X-ray spectroscopic and IR spectroscopic analyses of obtained experimental samples are given.

Data on the composition of industrial wastes and raw data of IR spectroscopic and X-ray spectral analyses samples of the construction materials are available in the Mendeley data repository [8]. These datasets are stored in the form of tables: the Table 1, Table 2, Table 3, Table 4 provide information on the chemical composition of waste, data in the Tables 5 and 6 are raw data for Figs. 2 and 3.

**Table 1:** Data of the composition of waste sulphur from the Zhanazhol deposit and the quality of standard sulphur of grades 9998-9920, (mass fractions in %).

Item	Grade 9998	Grade 9995	Grade 9990	Grade 9950	Grade 9920	Sulphur waste
1. Mass fraction of sulphur	99.2800	99.2500	99.9000	99.5000	99.200	99.06
2. Mass fraction of ash	0.02000	0.03000	0.05000	0.02000	0.04000	0.390
3. Mass fraction of organic substances	0.01000	0.03000	0.0600	0.02500	0.05000	0.53
4. Mass fraction of acid (by Н_2_SO_4_)	0.0015	0.0030	0.0040	0.0100	0.0200	0.02
5. Moisture content	0.200	0.200	0.200	0.200	0.00020	0.8
6. Mass fraction of arsenic	0.0000	0.0000	0.00000	0.0000	0.0300	less 10^-6^
7. Mass fraction of selenium	0.0000	0.0000	0.0000	0.0000	0.0400	traces
8. Mechanical impurities	None	None	None	None	None	−

**Table 2:** Monochromate sludge composition data.

Parameter	Quantity in dry substance, %	
	Minimum	Maximum
CrO_3_, w/s	0.23	0.38
Cr_2_O_3_, n/s	6.51	9.63
Fe_2_O_3_	17.00	19.00
Al_2_O_3_	8.00	12.00
SiO_2_	11.0	14.00
CaO	0.10	0.40
MgO	28.00	31.00
NaOH	1.36	4.98
H_2_O	0.10	0.14

**Table 3:** The data of the tailings chemical composition of the ferroalloy plant (average of three measurements).

Tailings	Chemical composition (analysis element)					
	TiO_2_	CaO	MgO	Al_2_O_3_	FeO_(gen.)_	SiO_2_
Sample No. 1	1.34	0.07	0.58	1.33	1.85	91.8
Sample No. 2	1.53	0.07	0.63	1.24	1.54	91.7

**Table 4:** Raw data on the composition of dry boron-containing slag - waste of a chemical plant.

Substance	CaO	MgO	B_2_O_3_	Fe_2_O_3_	Al_2_O_3_	SiO_2_	SO_3_
Concentration,%	31.47	1.933	0.194	0.323	16.29	6.78	43.01

**Table 5:** Raw data of the spectral composition of absorption bands of samples of chromate sludge and mastic.

Sample No. 1 Sludge		Sample No. 2 S-sludge	
Frequencies, cm^-1^	Type vibrations	Frequencies, cm^-1^	Type vibrations
604	ν_s_(Cr^3+^)	470	
666	ν_s_(Cr^3+^)	516	ν_s_(SO_4_)
871		771	ν_s_(Cr^3+^), ν(SO_4_)
967		800	ν_s_(Cr^3+^), ν_s_(SO_4_)
1108	ν_s_(SiO_2_)	1113	
1146		1617	
1622			
3395	ν_s_(SiO_2_)		
3532	ν(H_2_O)

**Table 6:** Raw data the chemical microanalysis of the phase surface of a sulphur concrete sample.

Element	Weighted %
Si	32.36
S	11.09
Ca	1.37
Fe	0.91

Table 1 of the repository shows data on the composition of waste sulphur from the Zhanazhol deposit and data on the quality of standard sulphur of grades 9998-9920 for their comparative analysis. The composition of lump sulphur samples (waste of oil and gas desulphurization of Zhanazhol field) has an increased content of impurities. At Zhanazhol field the radioactivity of sulphur, determined on dosimeter and on a recalculating unit for measuring radioactivity with a CTC-100 sensor measuring gamma radioactivity, was consistent with the field background.

In the Table 2 presents the data on the chemical composition of monochromate sludge of the chemical plant (Aktobe, Western Kazakhstan). The average samples are represented by silicates, clay minerals, carbonates among which magnesium minerals dominate over the others.

Table 3. Data on the chemical composition of the metallurgical waste from the Ferroalloy Plant (Aktobe, Western Kazakhstan).

Table 4. Raw data on the composition of dry boron-containing slag - waste from a chemical plant (Alga city, Western Kazakhstan). The presence of boron compounds in boron-containing waste can serve as a fire retardant, significantly affecting the fire resistance of sulphur in the composition of concrete.

The raw data for Fig. 2 of the spectral composition of the absorption bands of the chromate sludge and mastic samples are shown in Table 5 of the Mendeley Data repository.

The raw data for Fig. 3a,b of the X-ray spectral microanalysis of the phase surface of a sulphur concrete sample are shown in Table 6 of the Mendeley Data repository. The data in Table 1, Table 2, Table 3, Table 4 are averages of three measurements. The files in the repository are presented in an XLSX format.

## 3 Experimental Design, Materials and Methods

### 3.1 Obtaining an experimental composition of mastic and sulfur concrete

Experimental design began with the analysis and generation of data to investigate the possibility of using sulphur (hydrocarbon feedstock treatment waste) and various industrial waste as fillers and aggregates for the production of sulphur concrete, as well as determining the optimal compositions and identifying the most effective fillers and aggregates.

In this work the following materials were used as fillers: roasted pyrite from the chemical plant in Alga (Western Kazakhstan), dry boron-containing slag and monochromate sludge from the chemical plant in Aktobe (Western Kazakhstan), as well as tailings of a ferroalloy plant in the same city.

The technology for producing sulphur mastics was as follows: the calculated amount of sulphur and filler in the form of a dry mixture was placed in a drying oven (drying unit) and kept until the components reached a temperature of 140-150°C. The components studied were used in a finely ground state with a specific grain surface of 2500-3500 cm^2^/g. After that, mixing was carried out for 1-2 minutes until a homogeneous mass was obtained, and then the mixture was poured into heated metal molds located on a vibrating platform. After compaction, until the complete removal of air bubbles from the mass of the raw mixture, the samples were kept in molds until complete refrigeration at room temperature and were further tested. The data on the compositions and characteristics of the resulting sulphuric mastics are shown in Table 7. The data Table 7 shows that sulphur mastics based on roasted pyrite are stronger.

**Table 7:** Compositions, characteristics of sulphur mastics based on various wastes and physical, mechanical test data.

Components, wt.%					Characteristics of sulphur mastics		
Sulphur waste	Roasted pyrite	Boron-containing waste	Chromate sludge	Tailings	Medium density kg/m^3^	Compression strength MPa	Flexing strength, MPa
40	60				2300	35-40	12-14
40		60			2400	33-36	10-12
40			60		2400	35-38	10-12
40				60	2350	34-39	11-13

Properties of the developed composition were studied for experimental verification (Table 8).

**Table 8:** Composition and properties of experimental composition of sulphur concrete.

Components, properties	Quantity
Sulphur, wt. %	18
Boron-containing waste, wt. %	15
Slag (fr.015-5 mm), wt.%	67
Burning time, sec.	10
Ignition temperature, ^0^С	200
Weight loss, %	0.12
Frost resistance, cycles	150
Abrasion, g/cm^2^	0.15-0.20

The increased flame resistance of the suggested composition is explained by the presence of boron compounds in the waste composition, providing a more incombustible base.

The technical solution was achieved: a pre-dosed mixture of sulphur, aggregate, and filler was heated to a temperature of 140-150°C and mixed until it reached a homogeneous mass. Then the mixture was placed in metal molds preheated to a temperature of 130-140°C, installed on a vibrating table and compacted until intensive removal of air bubbles. After cooling to ambient air temperature, the test samples obtained strength. The quantitative content of the filler in each case was taken from the condition for the formation of compositions of the same viscosity. Concrete compositions were selected without traditional additives for various functional purposes – plasticizers and stabilizers. The following technology carried out production of sulphur concrete using the above industrial waste: a pre-dosed mixture consisting of sulphur, filler and aggregate was heated together to a temperature of 140-150°C and stirred to a homogeneous mass with different consumption of the composition components, mass. %:

Composition No. 1 (counting at 100%)

**Table:** 

Sulphur	17.5
Boron-containing waste	24.5
Quartz sand	18.5
Limestone mining waste	
Belogorsk field	39.5
Composition No. 2 (counting at 100%)	
Sulphur	18
Boron-containing waste	24
Quartz sand	19
Limestone mining waste	
Belogorsk field	39
Composition No. 3 (counting at 100%)	
Sulphur	18.5
Boron-containing waste	23.5
Quartz sand	19.5
Limestone mining waste	
Belogorsk field	38.5

Then the blended product was placed in in preheated to a temperature of 130-140°C metal forms mounted on a vibrating table and compacted until the intense removal of air bubbles. After cooling to ambient temperature, the samples of experimental batches obtained strength.

Quantitative content of the filler in each case was taken from the condition of formation of compositions of the same viscosity.

Production of sulphuric concrete using other above-mentioned industrial waste was carried out according to the following technology: a pre-dosed mixture consisting of sulphur, filler and aggregate was heated together to a temperature of 140-150°C and mixed until a homogeneous mass was obtained. Then the mixture was placed in metal molds preheated to a temperature of 130-140°C, mounted on a vibrating table and compressed until the intensive removal of air bubbles. After cooling to the ambient temperature, the test samples obtained strength.

### 3.2 Data on physical and mechanical properties of the obtained samples

The composition data and the results obtained for determining some of physical and mechanical properties of samples of experimental and control batches in comparison with GOST (Standard) are given in Table 9.

**Table 9:** Some physical and mechanical properties of sulphur concrete experimental and control batches.

Indicators	Control	Experimental batch No. 1	Experimen-tal batch No. 2	Experimen-tal batch No. 3	Concrete on dense aggregates (Standard 26633-2015)
Average density, kg/m^3^	2200	2400	2500	2450	1800-2000
Compressive strength, MPa	35	45	48	46	25
Flexing strength, MPa	12	13	15	14	-
Water absorption,%	3.0	2.5	2.0	2.5	No more than 5-7
Frost resistance, cycles /K_frs_	150	200/0.90	200/0.91	200/0.89	100

**Fig. 1:**
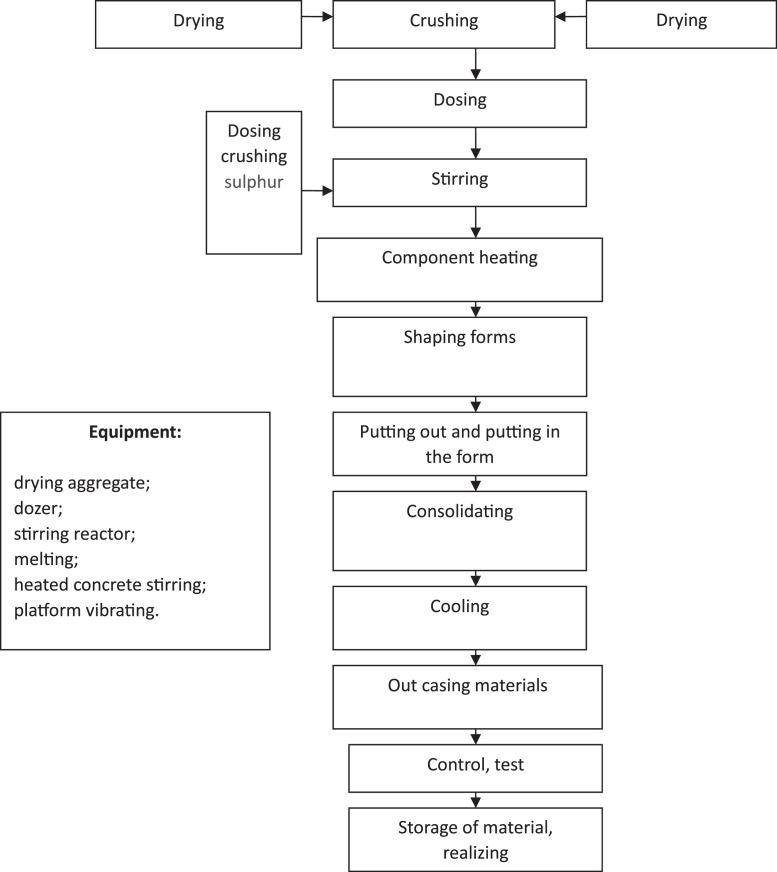
Experimental design-scheme construction material production with complex usage of wastes.

Process flow scheme for the production of sulphur-containing building materials with comprehensive use of waste materials is shown in Fig. 1.

Table 10 summaries the compositional data and indicators for the determination of the physical and mechanical properties of the test samples obtained from the wastes from the production facilities in the proximity of each other in the West of Kazakhstan.

**Table 10:** Components and physical and mechanical properties of sulphur concrete test samples.

Components, wt.%						Specifications		
Sulphur waste from oil and gas desulfurization, mass,%	Filler			Aggregate		Average density kg/m^3^	Compres-sion strength, MPa	Flexing strength, MPa
	Roasted pyrite waste, %	Chromate sludge, %	Tailings %	Quartz sand, %	Granite rubble waste, %			
18	26			18	38	2400	45-48	11-15
17		26		19	38	2300	42-45	11-13
19			28	20	33	2380	44-47	12-14

### 3.3 Thermodynamic calculation data

Reactions of interaction of sulphur with roasted pyrite can presumably proceed in two possible ways:

(1) $$\;2Fe_{2}O_{3}+\;7S\to \;4\text{FeS}\;+\;3\text{SO}{\;}_{2}↑$$

or:

(2) $$3S\;+\;2\text{FeO}\;\to \;2\text{FeS}\;+\;3SO_{2}↑$$

The probability to implement a process according to two above equations can be determined by thermodynamic calculations.

$$2Fe_{2}O_{3}+7S\to 4\text{FeS}+3SO_{2}↑$$

$$\begin{array}{ccccc} H_{298}^{0} & -822.16 & 0 & -100.42 & -296.90 \end{array}$$

(kJ/mol)

$$\begin{array}{ccccc} S_{298}^{0} & 87.45 & 31.92 & 60.29 & 248.07 \end{array}$$

(J/mol·K)

$$\begin{array}{ccccc} G_{0} & -740.34 & 0 & -100.78 & -300.21 \end{array}$$

(kJ/mol)

$$\begin{array}{ccccc} C_{p}^{0} & 103.76 & 22.68 & 50.54 & 39.87 \end{array}$$

(J/mol·K)

(3) $$\Delta H_{x.p.}^{0}=\Sigma H_{\text{fin}.}^{0}-\Sigma H_{\text{init}.}^{0}=4\times \left(-100.42\right)+3\times \left(-296.90\right)-2\times \left(-822.16\right)+0=351.94\;\text{kJ}/\text{mol}$$

$\Delta H_{x.p.}^{0}>0\left(\text{no}\;\text{reation}\right)$

(4) $$\Sigma G_{x.p.}^{0}=\Sigma G_{\text{fin}.}^{0}-\Sigma G_{\text{init}.}^{0}=4\times \left(-100.78\right)+3\times \left(-300.21\right)-2\times \left(-740.34\right)+0=176.93\;\text{kJ}/\text{mol}$$

$\Delta G_{x.p.}^{0}>0\left(\text{no}\;\text{reation}\right)$

(5) $$\Delta C_{p}^{0}=\Sigma C_{\text{fin}}^{0}-\Sigma C_{\text{init}}^{0}=3\times 39.87+4\times 50.54-2\times 103.76+7\times 22.68=-44.51\;J/\text{mol}\cdot K$$

(6) $$\Delta S_{x.p.}^{0}=\Sigma S_{\text{fin}.}^{0}-\Sigma S_{\text{init}.}^{0}=4\times 60.29+3\times 248.07-2\times 87.45+7\times 31.92=587.03\;J/\text{mol}\cdot K$$

$$\Delta S_{x.p.}^{0}>0.$$

If $\Delta S_{x.p.}^{0}>0$, then according to the II law of thermodynamics, the reaction should proceed, since it is accompanied by an increase in entropy. Apparently, the entropy effect increases its effect with increasing temperature. Let us calculate the value of ΔH^0^t at temperatures of 100^0^ and 150°C using the formula:

(7) $$\Delta H_{T}^{0}=\Delta H_{298}^{0}+\Delta C_{p}^{0}\left(T-298\right)$$

(8) $$\Delta H_{T}^{0}=351.94+\left(-44.51\times 100\right)\times 10^{-3}=347.49\;\text{kJ}/\text{mol}$$

(9) $$\Delta H_{T}^{0}=351.94+\left(-44.51\times 150\right)\times 10^{-3}=345.26\;\text{kJ}/\text{mol}$$

Reactions do not proceed even at increased temperatures, since $\Delta {Н}_{t}^{0}>\;0$.

The second variant:

(10) $$3S+2\text{FeO}\;\overset{t}{\to }\;2\text{FeS}+3SO_{2}↑$$

$$\begin{array}{ccccc} S_{298}^{0} & 31.92 & 60.29 & 60.29 & 248.07 \end{array}$$

(J/mol·K)

$$\begin{array}{ccccc} G_{298}^{0} & 0 & -244.30 & -100.78 & -300.21 \end{array}$$

(kJ/mol)

(11) $$\Delta G_{x.p.}^{0}=\Sigma G_{\text{fin}.}^{0}-\Sigma G_{\text{init}.}^{0}=2\times \left(-100.78\right)+\left(-300.21\right)-\left(-244.30\right)\times 2=-13.17\;\text{kJ}/\text{mol}$$

(12) $$\Delta S_{x.p.}^{0}=\Sigma S_{\text{fin}.}^{0}-\Sigma S_{\text{init}.}^{0}=2\times 60.29+248.07-3\times 31.92+2\times 60.75=151.39\;J/\text{mol}\cdot K$$

If the difference in thermodynamic potential $\left(\Delta G_{x.p.}^{0}\right)$ of substances involved in the reaction is less than zero, then there is a reaction.

Calculations show that an increase in temperature from 100°C to 150°C leads to a slight increase in the entropy factor. This indicates that at increased temperatures the difference in free energies $\left(\Delta G_{x.p.}^{0}\right)$ may have a negative value.

Indeed, the calculation of Δ*G*^0^ even at room temperature led to the fact that $\left(\Delta G_{x.p.}^{0}\right)$ becomes negative (-13.17 kJ/mol). This indicates the occurrence of a surface reaction, leading to an increase in sulphur adhesion to the surface of roasted pyrite grains.

Preliminary experiments have shown that a certain amount of roasted pyrite has properties of magnetization, which indicates the presence of $Fe_{3}O_{4}$, which includes $\text{FeO}\;(Fe_{3}O_{4}=\;\text{FeO}\;\times Fe_{2}O_{3})$.

### 3.4 IR spectroscopic analysis

In order to establish the nature of interaction of sulphur with components of wastes, using IR spectroscopic analysis, the structure of the initial raw materials and mastics based on them was studied.

**Fig. 2:**
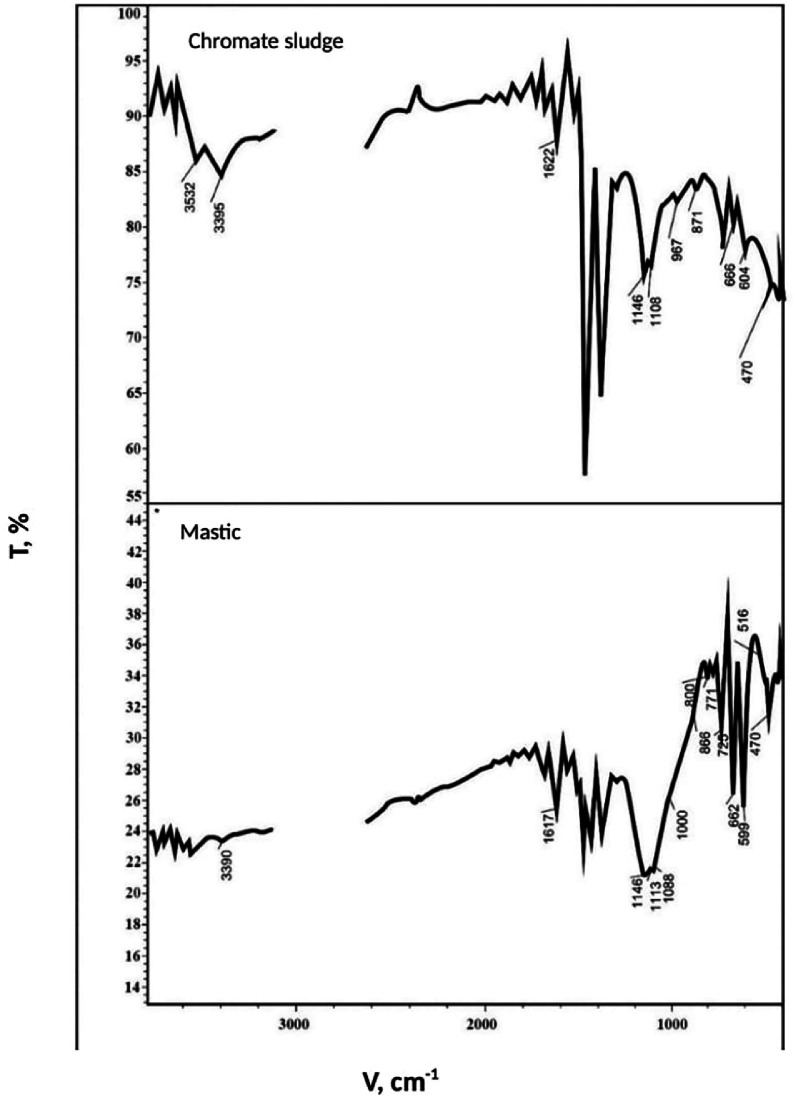
IR spectra of the samples.

Under the experimental conditions, sulphur had a coarse-crystalline structure, characteristic of rhombic modification. The injection of different components significantly changes the structure of sulphur and results in the production of a mastic with a fine-crystalline structure. These differences in structure are confirmed by IR spectroscopy data (Fig. 2).

Infrared spectra of the compositions were registered using a spectrophotometer in the region of wave numbers 400-4000 cm^−1^ from sample probs prepared as a suspension with vaseline oil.

Intensity of the main absorption bands in the IR spectra (Fig. 2) in the range of 604-666 cm^−1^ is typical for chromates, chromium (III) compounds, 1108-3395cm^−1^ - α⋅SiO_2_, possibly oxides. At the same time, microsections with absorption bands in the area of 516, 771, 800, 1113 cm^−1^ appear in the IR spectra of S-sludge (mastic), which, apparently, relate to neoplasms (sulfates) of the studied mastic.

The gap ($v=2750-3050\;cm^{-1}$) in the IR spectra of the starting material and mastic is explained by the fact that this region was not considered, as it is not informative and corresponds to valence vibrations of single associated water molecules.

Reducing the flammability of sulphur concrete is achieved by introduction of fire retardants, which allows to obtain compositions that tend to self-extinguish. There is a fact of introducing into the composition flame retardant additives that differ in aggregate state, dispersity, viscosity, for example, compounds of antimony, boron, phosphorus. It should be noted that the presence of boron compounds in the boron-containing waste can serve as a flame retardant that significantly affects the flame resistance of sulphur in the composition of concrete.

Monochromate sludge produced by a chemical plant when alloyed with sulphur significantly reduces its toxicity, due to the conversion of Cr (VI) to the less toxic form Cr (III) by the reaction:

(13) $$3S\;+\;2\text{Cr}\;\left(\text{VI}\right)\;\to \;Cr_{2}S_{3},$$

which is confirmed by the results of the above IR-spectroscopic study of structure of the mastic S-sludge.

### 3.5 Physicochemical analysis

**Fig. 3:**
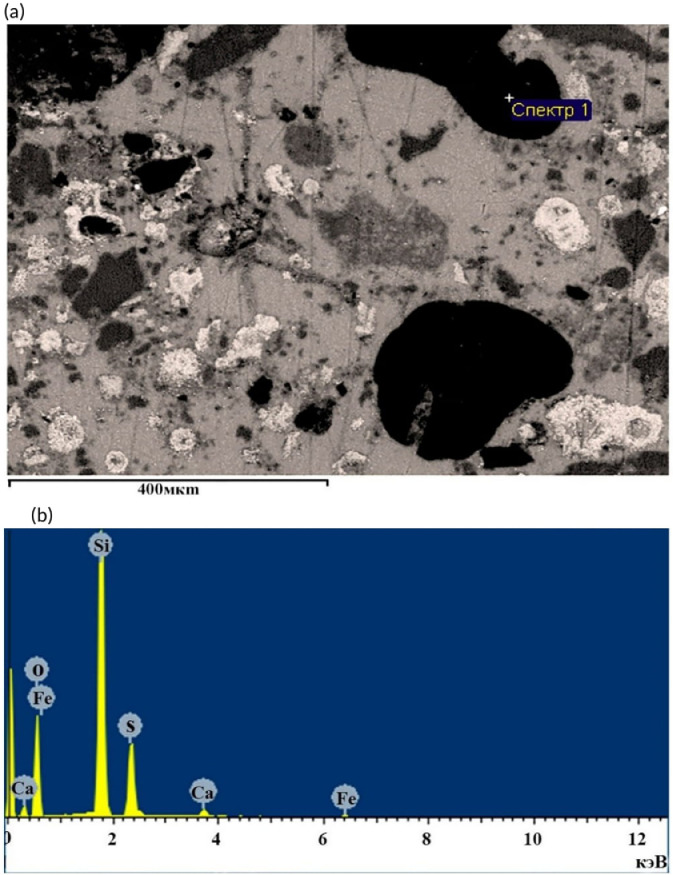
Microphotograph of the obtained prototype surface of sulphur concrete (a) and elemental composition of the phase surface (b).

Fig. 3(a) shows the microgeometry of sulphur concrete surface, which is obtained from waste (from Zhanajol sulphur, roasted pyrite, quartz sand and limestone mining waste) and 3(b) - the elemental composition, taken by the method of X-ray spectral analysis by registering topographic maps of the distribution of chemical element on the surface of the thin section.

Fig. 3(a) clearly shows the dense structure of sample, in which the particles are surrounded by shells (surface adhesion of sulphur), dark spots on some topographic sections of microsection are represented by resins and dots by silicon particles. The elemental composition of grinding surface (spectrum 1) is mainly consisted by compounds of silicon, iron and sulphur.

### 3.6 Data of indicative environmental and economic calculations

In order to determine the damage to the environment caused by chemical industry waste per year, in particular by monochromate sludge containing chromium (VI) and boric waste (using the example of Aktobe region), the following formula was used:

(14) y=γ×σ×M×A,

where γ - empirical coefficient, the numerical value of which is recommended to take equal to 4000 (units/cond. ton); σ - relative pollution hazard index (for Western Kazakhstan equal to 2.7); M - cumulative weight of the annual discharge of impurities (tons); A - relative toxicity index of the discharge or

(15) $$A=\frac{1\;(g/m^{3})}{\text{PEL}(g/m^{3})}$$

where PEL - is the permissible exposure limit of a substance equal to 0.02 mg/l for chromium (VI) and 0.017 mg/l for boron.

Mean annual content of the main pollutants downstream Ilek river, near Aktobe chromium plant was: for chromium (VI) - on average 20.3 PEL; for boron - on average 14 PEL. Since PEL of chromium (VI) is 0.05 mg/l for drinking water, and water flow rate in the river is 2.5 m^3^/sec, the value of annual damage (Y) will be equal to:

(16) $$\begin{array}{ccc} y_{\left(\text{Cr}\left(\text{VI}\right)\right)} & = & 4000\times 2.7\times 2.5\times 60\times 60\times 24\times 365\times \frac{1}{0,02}\times 20.3\times 0.02\times 10^{-6}\\ & = & 17.285\;\text{mln}.\text{rub}/\text{year}\left(\approx 302485\;\text{dollar}\right) \end{array}$$

Annual damage from the discharge of boron compounds will be equal to:

(17) $$\begin{array}{ccc} y_{\left(B\right)} & = & 4000\times 2.7\times 2.5\times 60\times 60\times 24\times 365\times \frac{1}{0.017}\times 13.8\times 0.017\times 10^{-6}\\ & = & 11.750\;\text{mln}.\text{rub}/\text{year}\left(\approx 205630\;\text{dollar}\right) \end{array}$$

Cumulative damage from combined chromium (VI) and boron contamination is $508116 per year.

The production cost of 1 m^3^ of sulphur concrete is 25-30% less than the same amount of cement-based concrete. Energy costs for the factory production of 1 m^3^ of sulphur concrete (without energy costs to produce binder) B-20 class is equal to 38 kg of fuel oil. Energy consumption for production of 1 m^3^ of cement concrete in precast concrete production reaches 164 kg of conditional fuel. The economy will therefore amount to 126 kg of fuel equivalent, due to the absence of heat and moisture treatment of sulphur composite materials, which is typical for cement-concrete products. The economic effect is also achieved as a result of releasing the factory space occupied by moulds and steaming chambers.

## Limitations

For the comprehensive recycling and joint processing of large-scale waste located in a wide geographical region (Western Kazakhstan), it was necessary to study and analyze waste of different compositions and types from different enterprises. Specifically: chrome-containing sludge from a chemical plant, waste from desulfurization of oil and gas raw materials from an oil refinery, roasted pyrite from sludge accumulators, production tailings of a ferroalloy enterprise, boron-containing slag from the territory of an inactive chemical plant. A large number of experimental research works have been performed in laboratories. Large-scale industrial tests were not carried out due to the lack of suitable equipment and space.

## Ethics Statement

This article does not contain any studies with human subjects, animal experiments, or any data collected from social media platforms. The datasets used in the article are open to the public. For the usage of these datasets, proper citation rules should be maintained.

## CRediT Author Statement

The work was performed without co-authors.

## Data Availability

Data of the industrial waste compositions in the Western Kazakhstan and raw data IR-spectroscopic, X-ray analysis of the samples construction materials (Original data) (Mendeley Data).
